# Clinical study on the difference in intestinal microecology between patients with preeclampsia and pregnant women at different stages of pregnancy

**DOI:** 10.3389/abp.2024.12020

**Published:** 2024-03-20

**Authors:** Fan Xie, Huan Zhang, Min Peng, TingTing Jiang

**Affiliations:** Department of Obstetrics and Gynecology, Maternal and Child Health Hospital of Hubei Province, Tongji Medical College, Huazhong University of Science and Technology, Wuhan, Hubei, China

**Keywords:** preeclampsia, lipid metabolism, renal function, intestinal flora, blood cell parameters

## Abstract

**Objective:** To explore the difference in intestinal microecology between patients with preeclampsia and pregnant women at different stages of pregnancy.

**Methods:** From January 2020 to January 2022, clinical data, including blood routine, lipid profile, and renal function indicators, were gathered from a cohort consisting of 5 cases of preeclampsia and 34 cases of non-preeclampsia. The non-preeclampsia group was further categorized into 6 cases in the First trimester, 13 cases in the Second trimester, and 15 cases in the Third trimester. The data collection took place at the Obstetrics Department of the Maternal and Child Health Hospital of Hubei Province. Additionally, fecal samples were obtained from each subject for 16S rDNA gene sequencing and subsequent analysis. The clinical data and composition characteristics of the gut microbiota in each group were analyzed, and the correlation between gut microbiota and clinical data was analyzed by the Spearman correlation analysis method.

**Results:** In comparison to pregnant women without preeclampsia, preeclampsia patients exhibited a statistically significant elevation in blood routine parameters (WBC, N, L, and PLT count), a rise in lipid-related indicators (TC, TG, and LDL-C levels), a reduction in HDL-C levels, and an increase in renal function-related indicators (Cr, BUN, UA and Pro levels). Compared with non-preeclampsia pregnant women, preeclampsia women exhibited an augmented diversity of gut microbiota. Differences in gut microbiota composition between the two groups were observed at the gate and genus levels. Moreover, there are significant differences in the composition of gut microbiota between the preeclampsia group and the third-trimester group in terms of genus and species, and this difference is mainly caused by *Prevotella* and *s_ Bacteroides*_ *Uniformis* and *Ruminococcus_ bromii*. In addition, actinobacteria, bifidobacterium at the genus level, and *Ruminococcus_bromii* at the species level are positively correlated with clinically relevant indicators (excluding HDL-C).

**Conclusion:** There are significant differences in gut microbiota between preeclampsia pregnant women and late pregnancy pregnant without preeclampsia, including *Prevotella* and *Bacteroides_ Uniformis,* and *Ruminococcus_ bromii*. In addition, these differential bacteria are correlated with most clinical indicators. However, additional comprehensive analysis is required to ascertain the functional correlation between these bacteria and clinical indicators.

## Introduction

Preeclampsia is a kind of hypertensive disorder complicating pregnancy (HDP) (Dimitriadis et al., 2023). The incidence of preeclampsia in pregnant women is 3%–5%, which is one of the main reasons for the increased mortality of pregnant women and perinatal infants (Arechvo et al., 2023; Hallum et al., 2023). Preeclampsia has adverse effects on the short-term and long-term health of pregnant women, including stroke, hypertension, and metabolic syndrome (Alanazi et al., 2022). Its offspring are prone to premature delivery, fetal distress, fetal growth restriction (FGR), neonatal hypoglycemia, and even death (Govender et al., 2023). The etiology of preeclampsia is multifactorial, including maternal factors such as family history of preeclampsia, multiple pregnancies, chronic kidney disease, and obesity, and placental factors such as uteroplacental insufficiency and increased placental volume/mass ratio (Syngelaki et al., 2022). However, the pathogenesis of preeclampsia has not been fully clarified. In addition to actively exploring effective treatment methods for preeclampsia and optimizing the prognosis, screening high-risk pregnant women with preeclampsia before pregnancy and taking targeted intervention measures may reduce the incidence of preeclampsia and fundamentally reduce the risk of pregnancy (Zheng et al., 2023).

In recent years, with the in-depth study of intestinal flora, it has been gradually found that changes in intestinal flora may be related to the onset of preeclampsia and affect the intestinal flora of offspring (Chen et al., 2020; Huang et al., 2022). The intestinal microbiota is a complex and huge microbial community living in the digestive tract, which can produce a variety of compounds that regulate the activities of remote organs and play an important role in host metabolism, immunity, and nutrition absorption (Ahmadian et al., 2020). Intestinal microorganisms promote the occurrence of insulin resistance by inducing the chronic inflammatory reaction of the host and causing the accumulation of fat by regulating the energy metabolism gene. Metabolic changes usually occur before clinical symptoms, so metabolic changes can be used as a marker to predict the occurrence and development of preeclampsia. Intestinal microorganisms and the human body form a “superorganism,” and the change and management of intestinal microbial structure are of great significance.

This study focuses on investigating the role of intestinal microflora in preeclampsia by comparing pregnant women with preeclampsia as the research group and healthy pregnant women as the control group. The study utilizes macro genome sequencing of stool samples to analyze the structure, species, functional composition, and metabolic pathways of the intestinal microflora. Furthermore, the study examines the potential significance of key species and gene functions in the development of preeclampsia, aiming to gain insights into its pathogenesis. Additionally, the study aims to provide theoretical support for the application of prevention and control strategies by predicting the macrogenome of intestinal microflora in preeclampsia.

## Materials and methods

### General information

This study collected 39 pregnant women recruited by the Maternal and Child Health Hospital of Hubei Province from January 2020 to January 2022. Inclusion criteria: 1) Filing for prenatal examination at 12^+6^ weeks of pregnancy; 2) Intrauterine singleton; 3) Age: 20–45 years old; 4) Regular prenatal examination, complete clinical data, and acceptable follow-up. Exclusion criteria: 1) hypertension and/or renal dysfunction were diagnosed before or during pregnancy; 2) Large gastrointestinal surgery before pregnancy; 3) History of antibiotics in the first 3 months of pregnancy; 4) Combined with acute and chronic gastrointestinal diseases and severe autoimmune diseases; 5) Complications included gestational diabetes, gestational heart disease, intrahepatic cholestasis and other serious complications of pregnancy. According to the guidelines for the diagnosis and treatment of hypertensive disorders during pregnancy, pregnant women who were diagnosed with preeclampsia after 20 weeks of pregnancy were regarded as the preeclampsia group (*n* = 5). Pregnant women without preeclampsia were divided into First-trimester (1–12 weeks of pregnancy, *n* = 6), Second-trimester (13–25 weeks of pregnancy, *n* = 13), and Third-trimester (26–40 weeks of pregnancy, *n* = 15). Age, pre-pregnancy body mass, and height were recorded, and pre-pregnancy body mass index (BMI) was calculated. The participants remained in a state of rest for 5 min, subsequently assuming a seated position, attentively relaxing their limbs, and selecting the cuff. The blood pressure of the right upper limb was measured, the cuff was at the same level as the heart, and the systolic blood pressure (SBP) and diastolic blood pressure (DBP) were recorded. The informed consent of all the subjects was signed by themselves or their families, and the ethics committee of the Maternal and Child Health Hospital of Hubei Province reviewed and approved the research (approval number: 20190911).

## Clinical data collection

### Determination of blood cell index

Peripheral blood (30 μL) was collected from the subjects’ fingers and mixed with EDTA anticoagulant. The red blood cell (RBC) count, white blood cell (WBC) count, neutrophil count (N), lymphocyte count (L), platelet (PLT) count, and hematocrit (HCT) were measured by SYSMEX 5 classification automatic blood cell analyzer. The sample shall be tested after the quality control is qualified.

### Collection and detection of serum samples

In the morning, 5 mL of elbow vein blood was collected from subjects on an empty stomach and centrifuged at 3,000 r/min for 10 min. The supernatant serum was collected, and total cholesterol (TC), triglyceride (TG), low-density lipoprotein cholesterol (LDL-C), high-density lipoprotein cholesterol (HDL-C), creatinine (Cr), urea nitrogen (BUN), and uric acid were measured by Beckman Coulter AU 5800 automatic biochemical analyzer.

### Urine sample collection and detection

A sample of 24-h urine was collected, and the first urine was discarded at 6 a.m. All urine after 24 h was collected the day before, including urine at 6 a.m. the next day, and the 24-h total urine output was recorded. Using a Hitachi 7180 biochemical analyzer, the pyrogallol red method was used to measure the quantity of urine protein per milliliter of urine. 24 h urine protein quantity = urine protein quantity per milliliter of urine × 24 h urine volume.

### Collection of stool samples and DNA extraction

Fresh fecal samples were collected from the subjects in sterile containers and immediately stored in liquid nitrogen. Following the standard protocol, 200 mg of each frozen fecal sample was obtained, and the genome was extracted using the QIAamp DNA Fecal Genomic DNA Extraction Kit (QIAGE, Germany). The concentration of the extracted DNA was determined using the NanoDrop 2000 spectrophotometer (Thermo Scientific, United States). The 16S rDNA V3 and V4 variable regions of all samples were amplified using the forward primer 338F (5′-ACC​TAC​GGG​GCA​G-3′) and the reverse primer (5′-GACTACHVGGGTWTCTAAT-3′).

### 16S ribosomal RNA (16S rRNA) amplicon pyrosequencing and microbial analysis

The products obtained from polymerase chain reaction (PCR) were quantified using a QuantiFluor ST fluorometer (Promega, United States). The mixture of PCR products was purified using a gel extraction kit provided by QIAGEN (Hilden, Germany). Illumina MiSeq instrument (Illumina, San Diego, California, United States) was utilized to establish a database, and data were assessed using fastqc (version 0.11.8) and multiqc (version 1.10). The metagenomic data underwent automatic low-quality data pruning and filtering using trim_galore (version 0.6.7). Bowtie (version 2.4.5) was used to compare RNA sequences with internal reference genes. Microbiome analysis was performed with Metapelan (version 3.0).

Usearch was used to cluster Tags with a similarity threshold of 97%. The α diversity (Shannon index) was determined using mother (version 1.39.1). Bray Curtis distance algorithm was used for principal component analysis (PCA). By dividing the number of sequences for phyla, genus, and species by the total number of sequences, the relative abundance was determined by applying normalization. The Linear Discriminant Analysis (LDA) method was used to evaluate the difference in species abundance among different samples. The threshold of LDA was 2. To facilitate a more comprehensive comparison of microbial communities, a phylogenetic tree was constructed using FastTree (ver2.1.9).

### Statistical analysis

All statistical analyses were performed using SPSS 21.0 software (SPSS Inc., Chicago, Illinois, United States) and the results were presented as mean ± standard deviation. The statistical significance of differences between two or three groups (or more) was determined using unpaired two-tailed t-tests or one-way ANOVA. The correlation analysis was conducted using Spearman correlation and the results were expressed as Spearman coefficient. The *p*-value of the Spearman correlation was adjusted using the FDR method. *p* < 0.05 was considered statistically significant. GraphPad Prism 8.0 software (GraphPad Software, Inc., La Jolla, CA, United States) was utilized for data visualization.

## Results

### General data comparison

**FIGURE 1:**
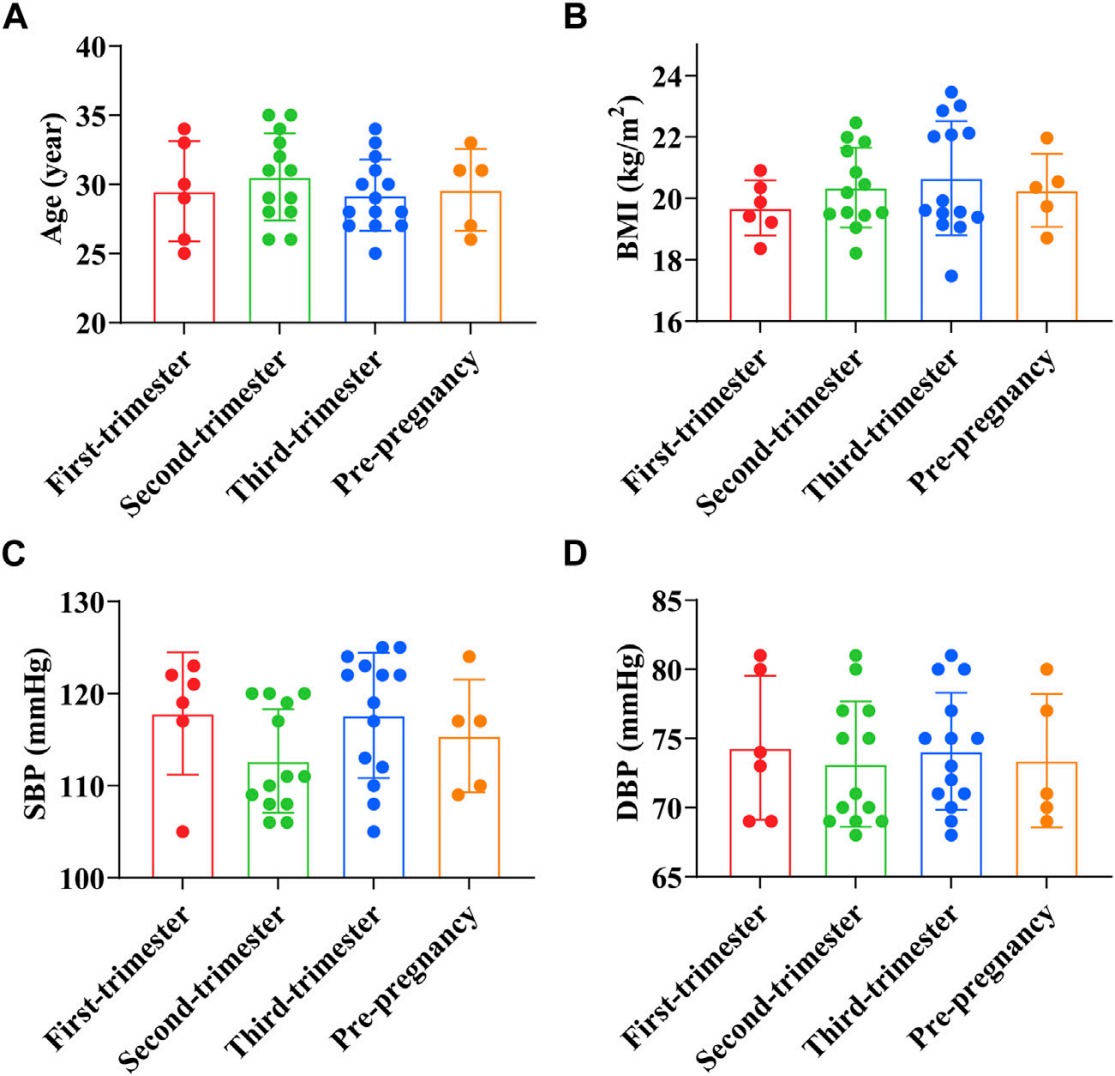
Comparison of general data of preeclampsia and pregnant women at different stages of pregnancy. **(A)** Age; **(B)** Pre-pregnancy body mass index; **(C)** Systolic blood pressure; **(D)** Diastolic blood pressure.

The age of pregnant women in the first trimester was (29.50 ± 3.62) years old, BMI before pregnancy was (19.69 ± 0.90) kg/m^2^, SBP was (117.83 ± 6.65) mmHg, and DBP was (74.33 ± 5.20) mmHg. The age of pregnant women in the second trimester was (30.50 ± 3.15) years old, BMI before pregnancy was (20.35 ± 1.30) kg/m^2^, SBP was (112.69 ± 5.62) mmHg, and DBP was (73.15 ± 4.54) mmHg. The age of pregnant women in the third trimester was (29.21 ± 2.58) years old, BMI before pregnancy was (20.66 ± 1.86) kg/m^2^, SBP was (117.64 ± 6.80) mmHg, and DBP was (74.07 ± 4.23) mmHg. The age of preeclampsia pregnant women was (29.6 ± 2.65) years old, BMI before pregnancy was (20.2 ± 1.06) kg/m^2^, SBP was (115.40 ± 5.46) mmHg, and DBP was (73.40 ± 4.31) mmHg. The results showed that age, BMI before pregnancy, and blood pressure of preeclampsia pregnant women were not significantly different from those of pregnant women at different stages of pregnancy (*p* > 0.05) (Figures 1A–D).

### Comparison of blood cell parameters

**FIGURE 2:**
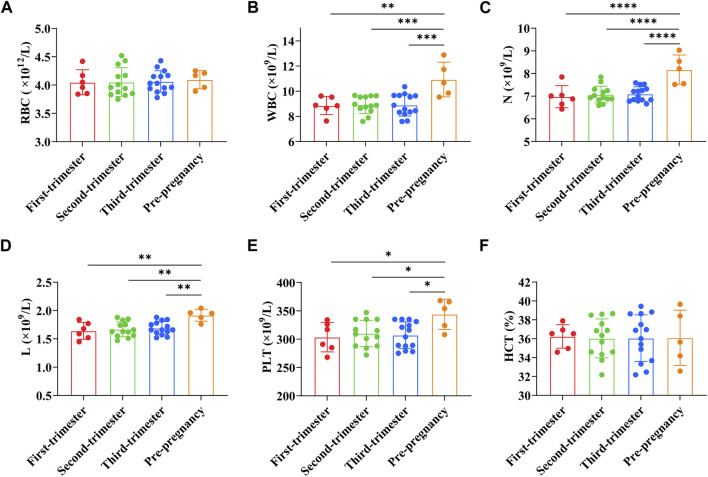
Comparison of blood cell parameters between preeclampsia and pregnant women at different stages of pregnancy. **(A)** Red blood cell count; **(B)** White blood cell count; **(C)** Neutrophil count; **(D)** Lymphocyte count; **(E)** Platelet count; **(F)** Hematocrit. *p < 0.05, **p < 0.01, ***p < 0.001, ****p < 0.0001.

RBC count of pregnant women in early pregnancy was (4.05 ± 0.22) × 10^12^/L, WBC count was (8.86 ± 0.72) × 10^9^/L, PLT count was (303.50 ± 25.99) × 10^9^/L, N count was (6.98 ± 0.49) × 10^9^/L, L count was (1.64 ± 0.15) × 10^9^/L, and HCT was (36.24 ± 1.24)%. RBC count of pregnant women in the second trimester was (4.05 ± 0.25) × 10^12^/L, WBC count was (8.92 ± 0.68) × 10^9^/L, PLT count was (310.00 ± 23.32) × 10^9^/L, N count was (7.07 ± 0.37) × 10^9^/L, L count was (1.67 ± 0.13) × 10^9^/L, and HCT was (36.04 ± 2.06)%. RBC count of pregnant women in the third trimester was (4.06 ± 0.19) × 10^12^/L, WBC count was (8.90 ± 0.87) × 10^9^/L, PLT count was (307.07 ± 23.47) × 10^9^/L, N count was (7.10 ± 0.32) × 10^9^/L, L count was (1.69 ± 0.12) × 10^9^/L, and HCT was (36.06 ± 2.47)%. RBC count of preeclampsia pregnant women was (4.09 ± 0.14) × 10^12^/L, WBC count was (10.93 ± 1.23) × 10^9^/L, PLT count was (344.00 ± 23.90) × 10^9^/L, N count was (8.17 ± 0.58) × 10^9^/L, L count was (1.92 ± 0.09) × 10^9^/L, and HCT was (36.10 ± 2.62)%. The results showed that the WBC count, N count, L count, and PLT count of preeclampsia pregnant women were significantly higher than those of pregnant women at different stages of pregnancy (*p* < 0.01, *p* < 0.001, *p* < 0.0001, *p* < 0.05) (Figures 2A–F).

### Comparison of serum lipid indexes

**FIGURE 3:**
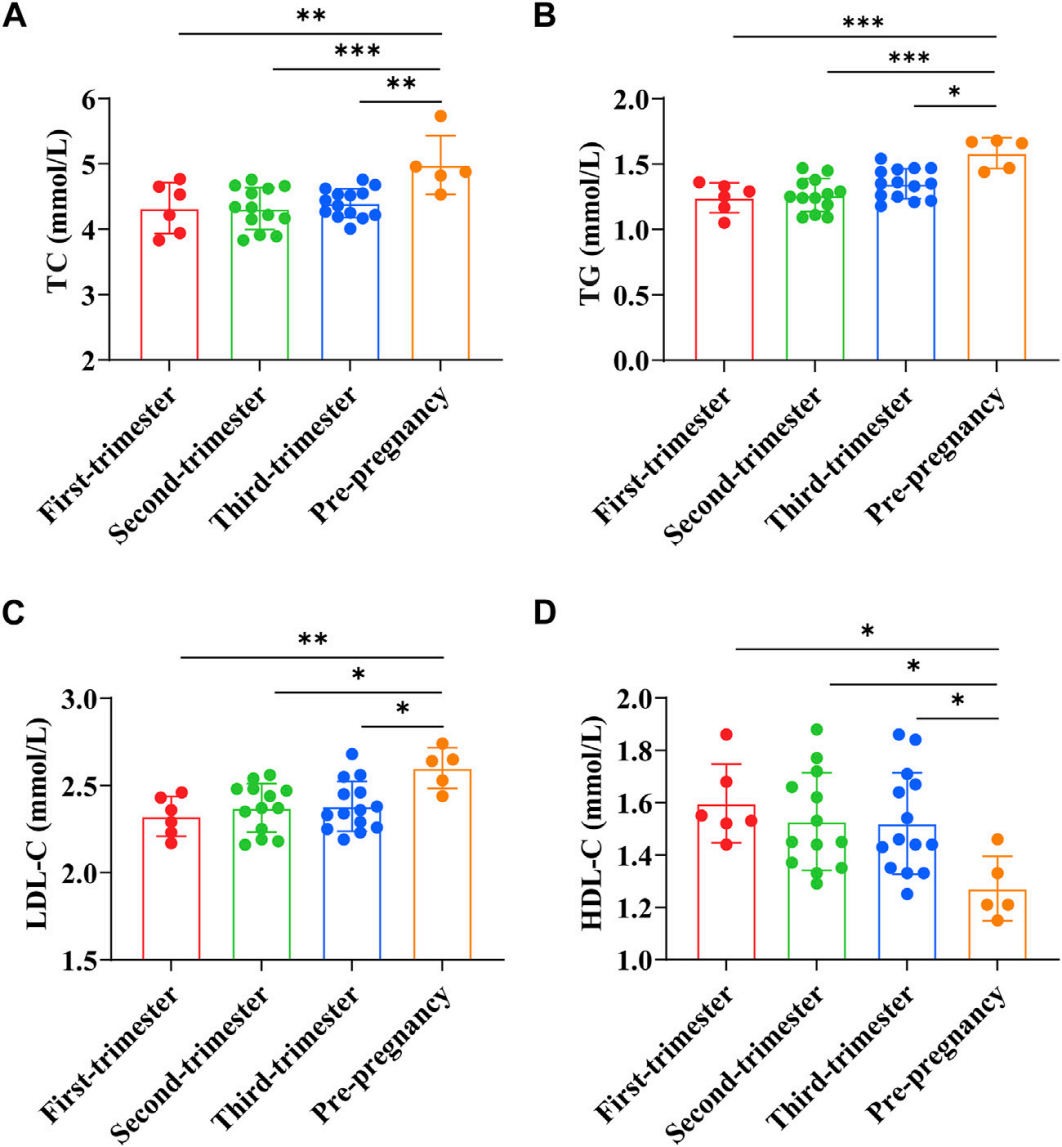
Comparison of serum lipid indexes between preeclampsia and pregnant women at different stages of pregnancy. **(A)** Total cholesterol; **(B)** Triglyceride; **(C)** Low-density lipoprotein cholesterol; **(D)** High-density lipoprotein cholesterol. *p < 0.05, **p < 0.01, ***p < 0.001, ****p < 0.0001.

TC, TG, LDL-C, and HDL-C were (4.32 ± 0.39) mmol/L, (1.24 ± 0.11) mmol/L, (2.32 ± 0.11) mmol/L, and (1.60 ± 0.15) mmol/L, respectively in early pregnancy. TC, TG, LDL-C, and HDL-C were (4.32 ± 0.32) mmol/L, (1.26 ± 0.13) mmol/L, (2.37 ± 0.14) mmol/L, and (1.53 ± 0.19) mmol/L, respectively in the second trimester of pregnancy. TC, TG, LDL-C, and HDL-C were (4.40 ± 0.22) mmol/L, (1.35 ± 0.11) mmol/L, (2.38 ± 0.14) mmol/L, and (1.52 ± 0.19) mmol/L, respectively in the third trimester of pregnancy. In preeclampsia pregnant women, TC was (4.98 ± 0.40) mmol/L, TG was (1.58 ± 0.11) mmol/L, LDL-C was (2.60 ± 0.10) mmol/L, and HDL-C was (1.27 ± 0.11) mmol/L. The results showed that the levels of TC, TG, and LDL-C in preeclampsia women were significantly higher than those in different stages of pregnancy (*p* < 0.01, *p* < 0.001, *p* < 0.0001, *p* < 0.05), while the levels of HDL-C were significantly lower (*p* < 0.05) (Figures 3A–D).

### Comparison of renal function indicators

**FIGURE 4:**
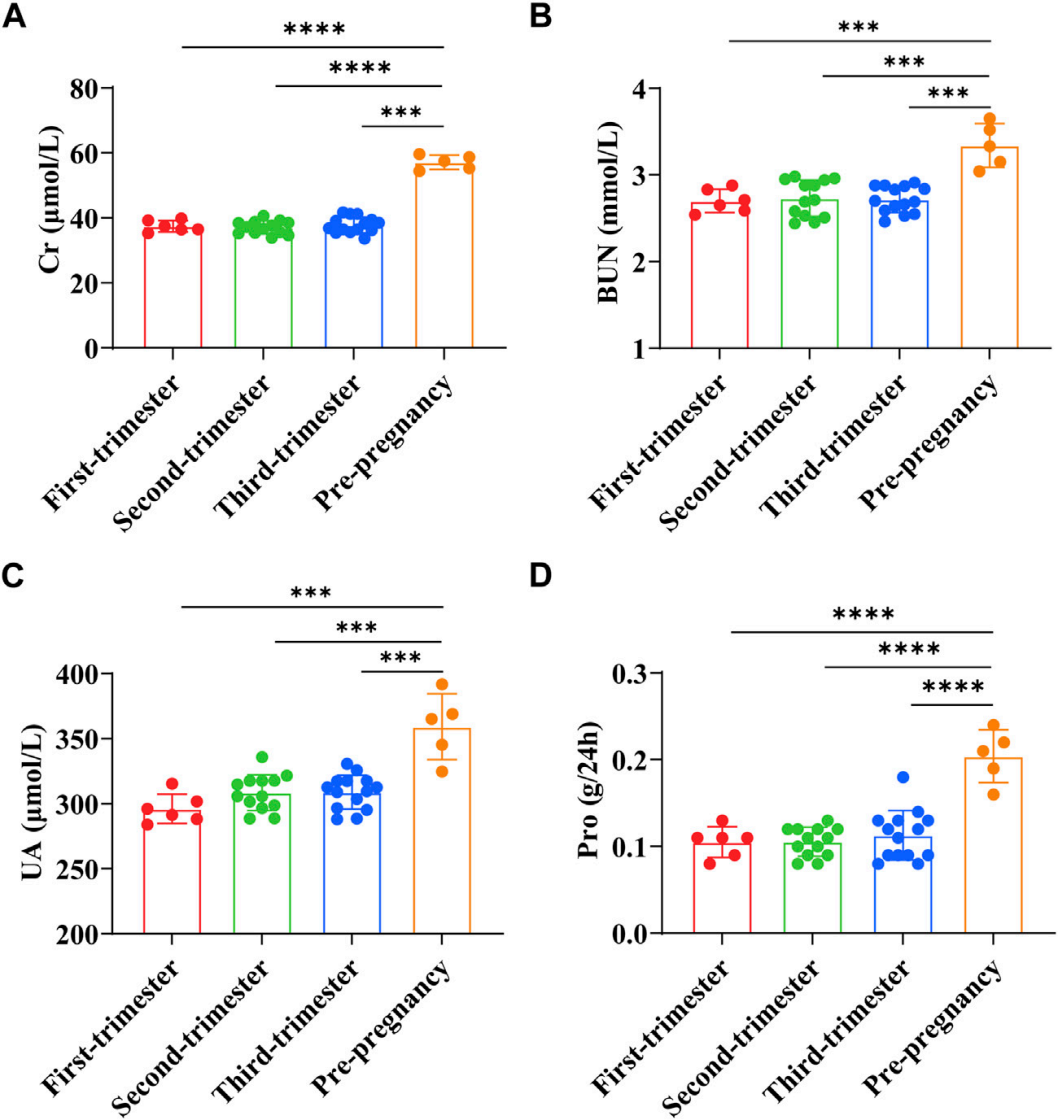
Comparison of renal function indexes between preeclampsia and pregnant women at different stages of pregnancy. **(A)** Creatinine; **(B)** Urea nitrogen; **(C)** Uric acid; **(D)** Urine protein. ****p < 0.0001.

Cr of pregnant women in early pregnancy was (37.43 ± 1.74) μmol/L, BUN was (2.70 ± 0.13) mmol/L, UA was (296.13 ± 11.27) μmol/L, and Pro was (0.11 ± 0.02) g/24h. Cr of pregnant women in the second trimester was (37.05 ± 2.02) μmol/L, BUN was (2.73 ± 0.21) mmol/L, UA was (308.50 ± 13.82) μmol/L, and Pro was (0.11 ± 0.02) g/24h. Cr of pregnant women in the third trimester was (37.85 ± 2.46) μmol/L, BUN was (2.72 ± 0.15) mmol/L, UA was (308.86 ± 13.03) μmol/L, and Pro was (0.11 ± 0.03) g/24h. Cr of preeclampsia pregnant women was (57.10 ± 1.97) μmol/L, BUN was (3.34 ± 0.23) mmol/L, UA was (359.14 ± 22.64) μmol/L, and Pro was (0.20 ± 0.03) g/24h. The results showed that the levels of Cr, BUN, UA, and Pro in preeclampsia women were significantly higher than those in different stages of pregnancy (*p* < 0.0001) (Figures 4A–D).

### Analysis of flora results

**FIGURE 5:**
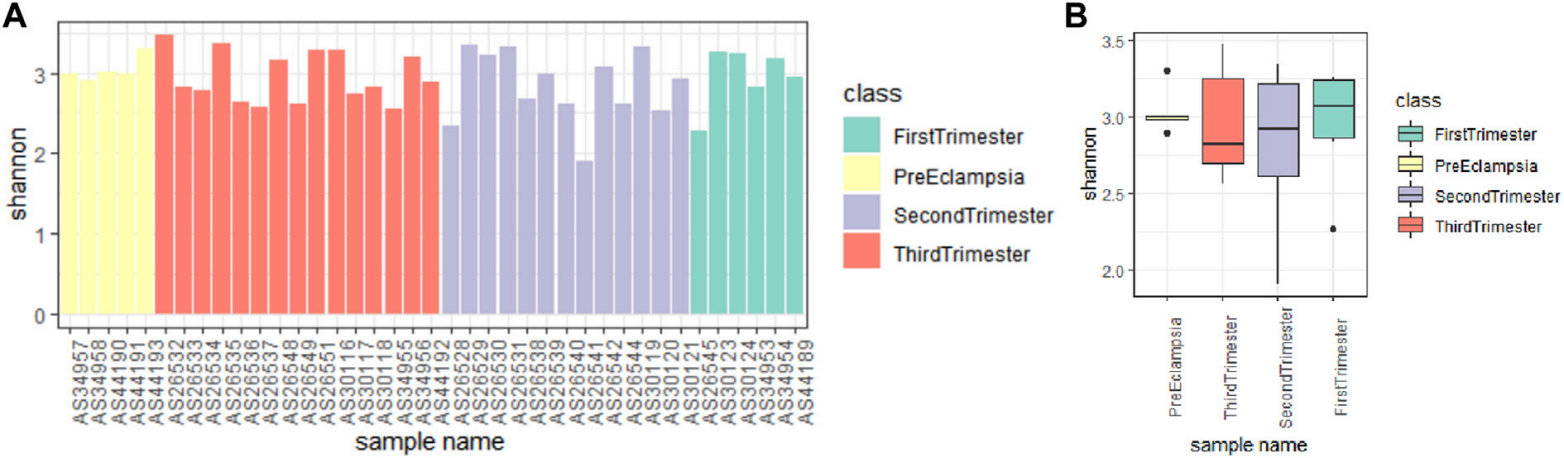
Alpha diversity analysis of microbial communities in different stages of pregnancy and preeclampsia. **(A)** Shannon index analysis of each sample; **(B)** Compare the Shannon index analysis of samples between groups. The Shannon index reflects the microbial composition. A higher Shannon index signifies a greater level of community diversity.

By observing the data quality of the original data, it was found that the sequencing data of 39 fecal samples were normal, and most of the sequencing readings were above q30, so there was no need to filter the samples. Shannon index can reflect the species diversity of the community. As shown in Figures 5A, B, the preeclampsia group and the First-trimester group had a higher diversity of intestinal flora. In addition, the gradual progression of the pregnancy cycle appears to be associated with a gradual decline in the diversity of the gut microbiota of pregnant women (Figures 5A, B).

**FIGURE 6:**
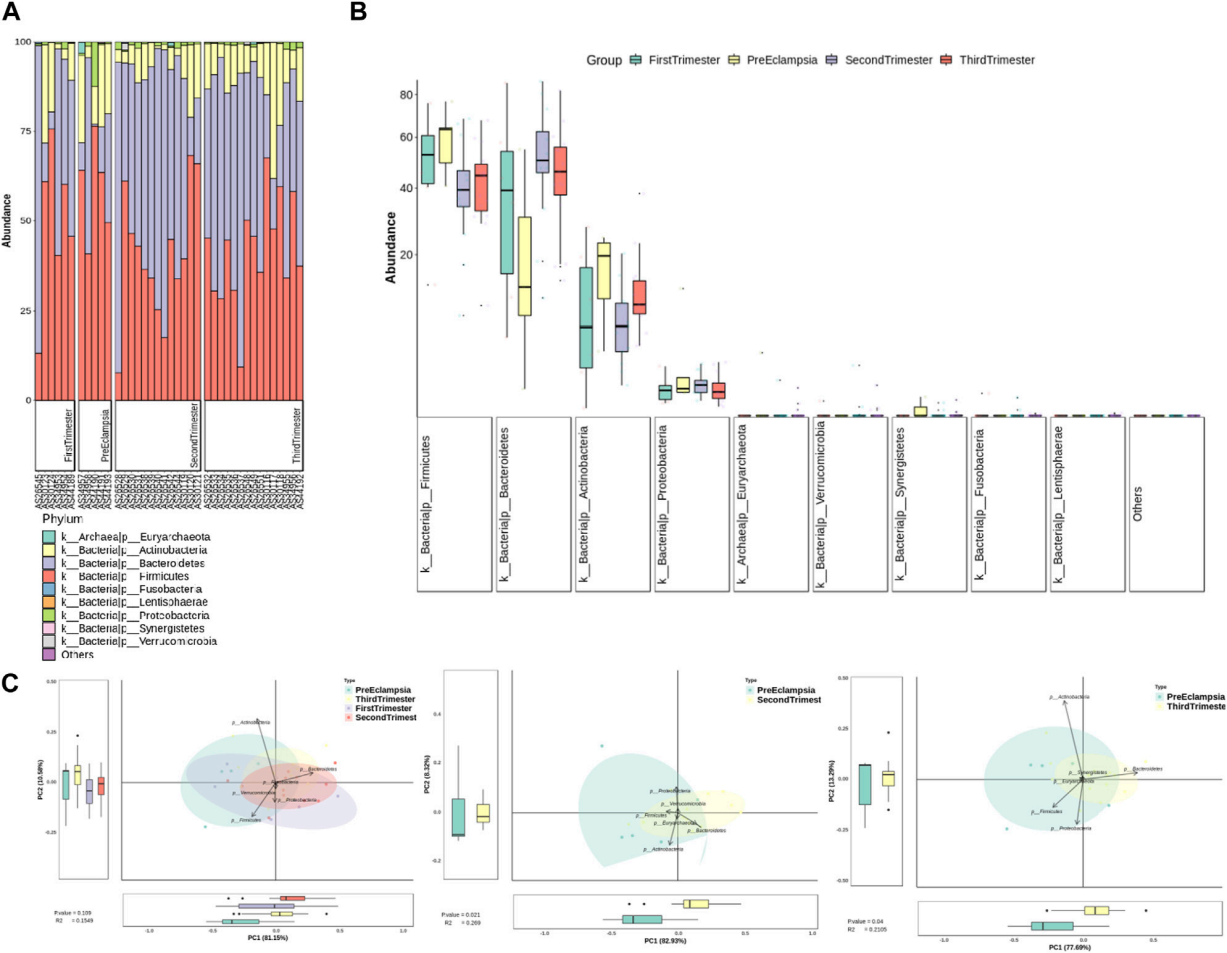
Analysis of bacterial flora level in different stages of pregnancy and preeclampsia. **(A)** Microbial distribution of each sample at the phylum level; **(B)** The distribution of the top 9 microorganisms at the phylum level in each group; **(C)** Differences in inter-group microbiota at the phylum level of principal component analysis (PCA).

At the gate level, the bar chart and the box chart were drawn with the relatively abundant phylum. The results showed that there were significant differences in the levels of *Bacteroides* and Actinobacteria between preeclampsia and other gestational periods, and the differences were more obvious between preeclampsia and other pregnancy periods (Figures 6A–C).

**FIGURE 7:**
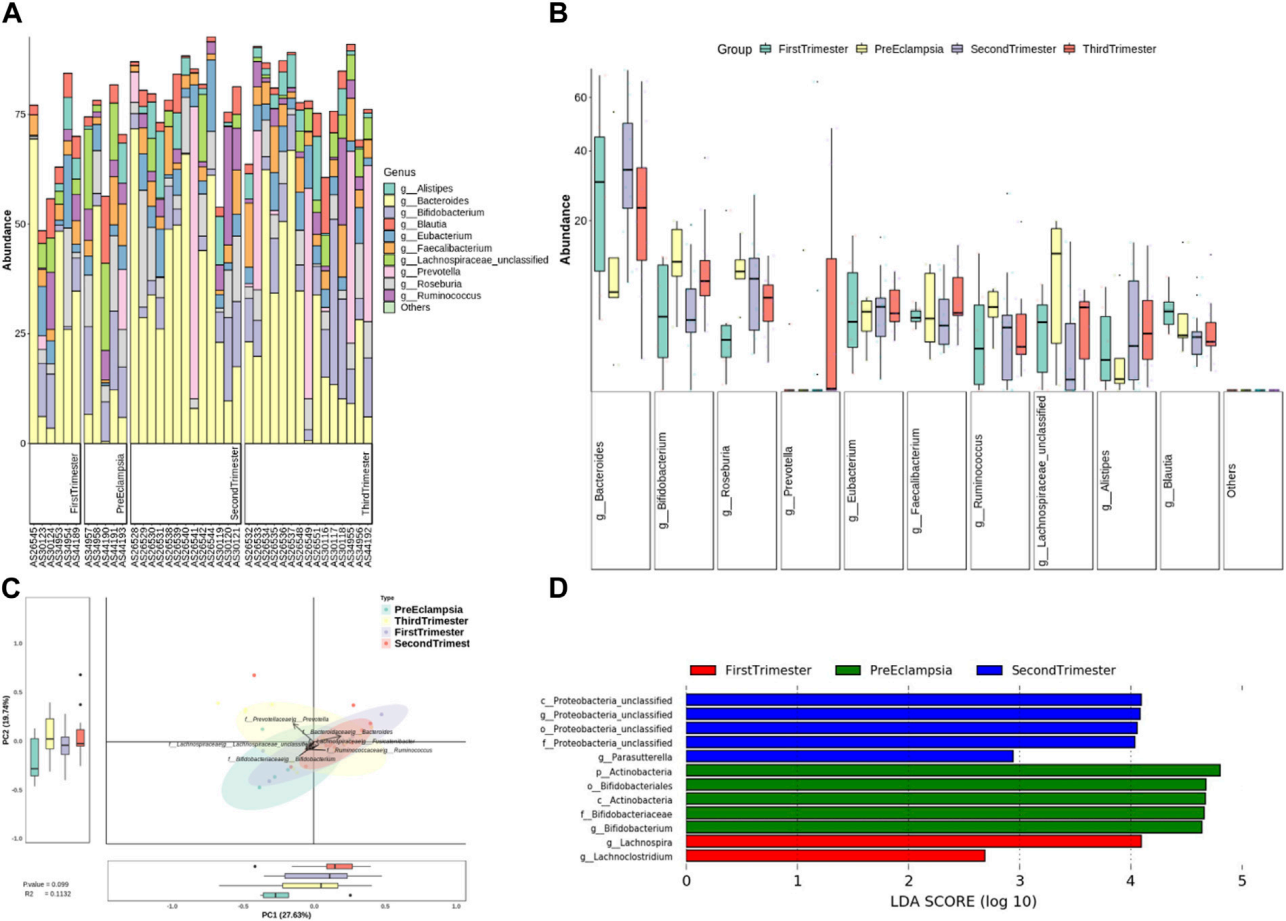
Analysis of bacterial flora level in different stages of pregnancy and preeclampsia. **(A)** Microbial distribution of each sample at the genus level; **(B)** The distribution of the top 10 microorganisms at the genus level in each group; **(C)** Differences in inter-group microbiota at the genus level of PCA; **(D)** Differential taxa between groups were identified using the LEfSe analysis at a threshold of 2.

At the genus level, the bar chart and box chart were drawn with the relatively abundant bacteria. The results showed that the level of *Bacteroides* and *Bifidobacterium* in preeclampsia and other pregnancy periods was significantly different (Figures 7A, B). In addition, compared with other pregnancy periods, *Lachnospiracea* increased in preeclampsia (Figures 7A, B). The PCA diagram at the generic level also showed that the difference between preeclampsia and late pregnancy was the most obvious in the overall difference comparison of each group, and the difference was mainly caused by *Prevotella* (Figure 7C). According to LDA, there were significant differences in abundance between different groups. At the genus level, the microbial community structure of the First-trimester group was mainly represented by *lachnospira* and *lachnochlostritium* under the phylum Firmicutes. The microbial community structure of the Second-trimester group was mainly represented by unclassified Proteobacteria under the phylum *Proteobacteria*; The microbial community structure of the preeclampsia group was mainly represented by *Actinobacteria* and *Bifidobacterium* under Phylum Actinobacteria (Figure 7D).

**FIGURE 8:**
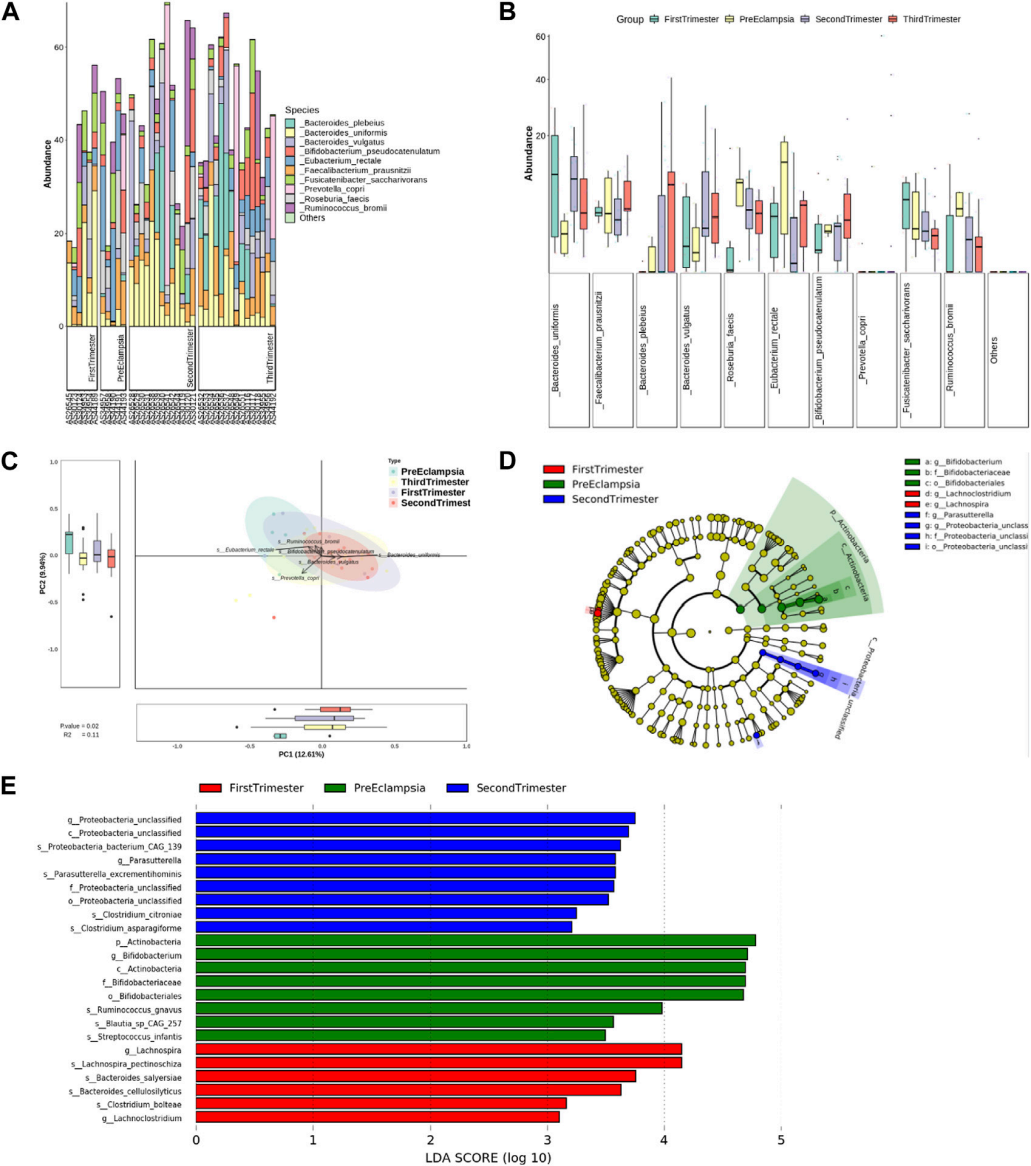
Analysis of bacterial flora level in different stages of pregnancy and preeclampsia. **(A)** Microbial distribution of each sample at the species level; **(B)** The distribution of the top 10 microorganisms at the species level in each group; **(C)** Differences in inter-group microbiota at the species level of PCA; **(D)** Phylogenetic tree analysis; **(E)** at the species level, differential taxa between groups were identified using the LEfSe analysis at a threshold of 2.

At the species level, the bar chart and box chart were also drawn with the top 10 species of relative abundance. The results showed that the abundance of *Bacteroides_uniforms* was relatively low in preeclampsia, while the abundance of Eubacterium Rectale was relatively high (Figures 8A, B). The PCA diagram at different levels also showed that in the comparison of the overall differences among the groups, the differences between preeclampsia and late pregnancy were obvious, and the main causes of the differences were *Bacteroides_uniforms* and *Ruminococcus_ bromii* (Figures 8C, D). Based on the LDA, at the species level, the microbial community structure in the First-trimester group was predominantly characterized by the presence of *Lachnospira_pentinoschiz*a, *Bacterodies_salyersiae* and *Bacterodies_cellulosilyticus*. In the Second-trimester group, the bacterial genus structure was represented by *Proteobacteria_bacterium_CAG_139*, *Parasutterella_excrementihomins*, *Clostridium_citroniae,* and *Clostridium_asparagiforme*. Conversely, the species structure of the preeclampsia microbiota was predominantly represented by *Ruminococcus_gnavus*, *Blautia_sp_CAG_257*, and *Streptococcus_infantis* (Figure 8E).

### Correlation analysis

**FIGURE 9:**
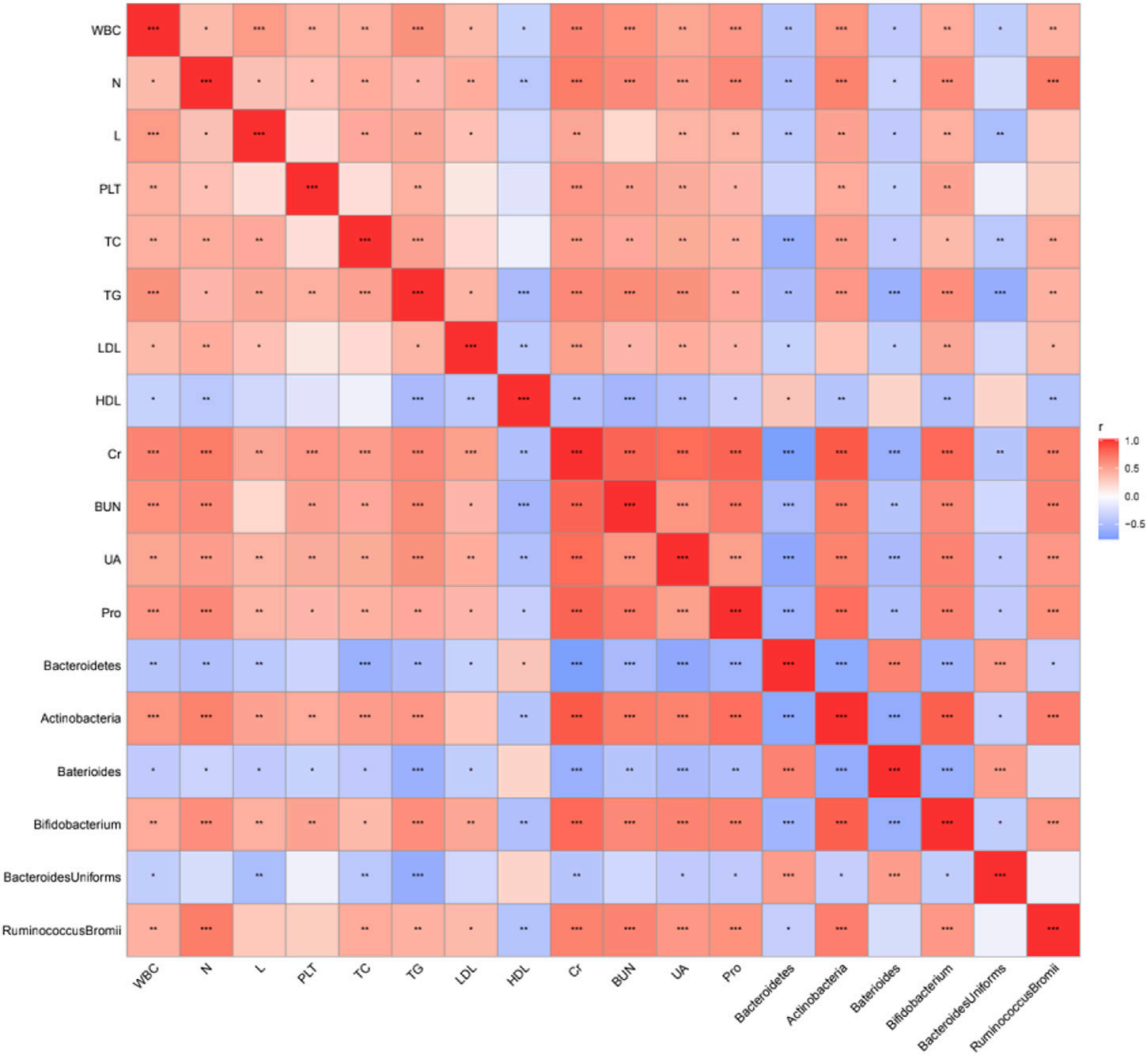
Correlation analysis between differential bacteria and clinical data. *p < 0.05, **p < 0.01, ***p < 0.001, ****p < 0.0001.

The correlation between different grades of bacteria and clinical data was analyzed. The results showed that *Bacteroides* at the gate level (except HDL-C) and *Bacteroides* at the genus level were negatively correlated with all clinically relevant indicators (*p* < 0.05, *p* < 0.01, *p* < 0.001), while *Bacteroides* at the gate level was positively correlated with HDL-C level (*p* < 0.05) (Figure 9). *Bacteroides_uniformis* at different levels were negatively correlated with WBC, TC, TG, Cr, UA, and Pro (Figure 9). In addition, Actinomycetes at the phylum level, *Bifidobacteria* at the genus level, and *Ruminococcus_bromii* at the species level were positively correlated with almost all clinically relevant indicators (except HDL-C) (*p* < 0.05, *p* < 0.01, *p* < 0.001), and negatively correlated with HDL-C level (*p* < 0.01) (Figure 9).

## Discussion

Preeclampsia is a metabolic syndrome related to pregnancy (Udenze, 2016; Hodgman et al., 2022). Hypertension and proteinuria occur after 20 weeks of pregnancy, which is the main cause of morbidity and mortality of pregnant women and perinatal infants. Its pathogenesis and its relationship with obstetric complications is a matter of concern today (Litwinska et al., 2021). At present, there is no reliable, effective, and economic screening method to predict preeclampsia (Khan et al., 2020). Studies have shown that some hematological changes occur during pregnancy, such as preeclampsia. The pathogenesis of preeclampsia is divided into two consecutive stages. Among them, the first stage occurs at the maternal-fetal junction. Due to the insufficient infiltration of trophoblast cells in the uterine wall and spiral artery, the blood flow of the uteroplacental artery is reduced, and the placental perfusion is insufficient, resulting in oxidative stress and placental dysfunction. This hypoxia state can induce inflammation by releasing chemokines, promoting cytokines, anti-angiogenesis factors, and neutrophils. The second stage begins with the infiltration of activated neutrophils into the maternal vascular tissue and is related to maternal systemic vascular inflammation, which can lead to vasoconstriction, hypertension, endothelial dysfunction, and end-organ ischemia. The above conditions will lead to the increase of white blood cells and neutrophils in the peripheral blood of preeclampsia pregnant women (Gogoi et al., 2019; Lu and Hu, 2019). A blood routine is a simple and practical test item, in which RBC, WBC, PLT, N, and L are common peripheral blood parameters in blood routine. The results of this study showed that the WBC count, N count, L count, and PLT count of preeclampsia pregnant women were significantly higher than those of pregnant women at different stages of pregnancy. The main reason for the increase of WBC during pregnancy is the increase of granulocytes caused by bone marrow proliferation and the left shift of the nucleus, that is, more immature WBC appears in the blood circulation (Awor et al., 2023). Although the number of WBCs increased, the chemotaxis and adhesion function of WBCs decreased from the second trimester of pregnancy. The inhibition of WBC function during pregnancy was related to the inhibition of humoral-cellular immune regulation function. Due to the poor ability of N denaturation, WBC is detained in the capillary in the low perfusion area of the thrombosis site, which releases lysosomes, histamines, leukotrienes, and other substances that are harmful to blood flow, resulting in microcirculation blood flow disorder (Moodley et al., 2020; Abdelzaher et al., 2022). The etiology theory of hypertensive disorder complicating pregnancy also believes that the abnormal immune regulation function and the toxic effect of oxidative stress reaction ultimately lead to N inflammatory infiltration.

The process of lipid metabolism *in vivo* is complex, and its parameters are risk factors for the occurrence and development of coronary heart disease, atherosclerosis like artery Congee, hypertension, and other diseases (Negre-Salvayre et al., 2022; Salma, 2022). Research shows that with the growth of pregnancy, the blood lipid level of normal pregnant women will increase (Vaught et al., 2023). The change in blood lipids in pregnant women is considered an adaptive change to support fetal development, but abnormal blood lipids will lead to adverse pregnancy outcomes including preeclampsia (Yang et al., 2022). The results of this study show that the serum lipid level of preeclampsia pregnant women were higher than that of other pregnancy periods, suggesting that the changes in serum lipid in early pregnancy have certain predictive value for preeclampsia. The lipid peroxidation of pregnant women is significantly enhanced, which can further promote the production and secretion of inflammatory factors, cause vascular endothelial damage, and promote the occurrence and development of preeclampsia (Phoswa and Khaliq, 2021).

Creatinine is mainly filtered by glomerulus and not reabsorbed by renal tubules (Tesfa et al., 2022; Wind et al., 2022). It is a good indicator of glomerular filtration function. Serum uric acid is the product of purine metabolism in the body, and the physiological concentration of uric acid has a positive effect on the body (Atigan et al., 2022). For pregnant and lying-in women, uric acid concentration is affected by continuous physiological changes during the whole pregnancy. In early pregnancy, due to the increase in blood volume and glomerular filtration rate, serum uric acid decreases by about 25%–35%. In the second trimester of pregnancy, the serum uric acid concentration begins to rise and is close to or even higher than the uric acid value of non-pregnant women at term. Research shows that the sudden increase in serum uric acid concentration may be related to the development of potential hypertension, and the adverse outcome of preeclampsia patients is more related to high uric acid concentration (Yu et al., 2023). In addition, a large amount of proteinuria can lead to hypoproteinemia, which can promote the formation of pleural effusion and ascites in severe cases, and affect respiration function in pregnant women. At the same time, hypoproteinemia can also stimulate the mother to increase the synthesis of lipids and lipoproteins (Malz, 1946). Hyperlipidemia can cause placental atherosclerosis. In addition, the spasm of placental arterioles can increase the blood flow resistance, and the placental blood flow perfusion is insufficient, putting the fetus in a chronic hypoxia state for a long time. It can also cause fetal intrauterine malnutrition, fetal growth restriction, fetal distress, and neonatal asphyxia. Therefore, the continuous occurrence of urinary protein will lead to further aggravation of preeclampsia, which can form a vicious circle. The results of this study showed that the levels of Cr, BUN, UA, and Pro in preeclampsia pregnant women were significantly higher than those in pregnant women at different stages of pregnancy, suggesting that abnormal renal function may be the influencing factor for the occurrence and development of preeclampsia.

In order to adapt to the physiological state during pregnancy and provide a good growth and development environment for the fetus, some metabolic changes will occur in the mother, and the abundance and diversity of intestinal flora will also change significantly during pregnancy, including preeclampsia. Intestinal flora can secrete inflammatory factors and anti-inflammatory factors, which are in a stable balance. Once the flora is out of balance, the balance will be destroyed, and inflammatory factors will increase, which will damage vascular endothelial cells, and then affect the stability of the blood system. *Bacteroides*, Bifidobacterium, and Lachnospiraceae have been proven to be related to the inflammatory-immune response mediated by intestinal flora (Miao et al., 2021; Huang et al., 2022; Lv et al., 2022). In addition, dyslipidemia and renal dysfunction are also important factors affecting the occurrence and development of preeclampsia. Bifidobacterium and Lachnospiraceae have been proven to be related to dyslipidemia and renal dysfunction (Sun et al., 2020). This is consistent with the results of this study. In our study, we observed a decrease in the abundance of *Bifidobacteria* and an increase in the abundance of *Lachnospiraceae* within the preeclampsia group. Furthermore, these patients exhibited abnormal blood lipid profiles and renal dysfunction.

To sum up, compared with pregnant women at different stages of pregnancy, the blood routine-related indicators (WBC count, N count, L count, and PLT count) of preeclampsia pregnant women were significantly increased, the blood lipid-related indicators (TC, TG, and LDL-C levels) were significantly increased, while the HDL-C level was significantly decreased, the renal function-related indicators (Cr, BUN, UA, and Pro levels) were significantly increased, the alpha diversity was relatively high, and the flora was relatively rich, which was the largest difference from late pregnancy. Among them, *Bacteroides* and actinomycetes have great differences at the phylum level, *Bacteroides* and *Bifidobacteria* have great differences at the genus level, *Bacteroides uniformis* and *Ruminococcus Bromii* have great differences at the species level, and the difference bacteria have a correlation with the relevant indicators of pregnancy. However, a more extensive sample size is necessary to conduct further investigation into these findings, particularly in relation to the outcomes of gut microbiota.

## Data Availability

The datasets presented in this study can be found in online repositories. The names of the repository/repositories and accession number(s) can be found in the article/supplementary material.

## References

[B1] Abdelzaher W. Y. Mostafa-Hedeab G. Bahaa H. A. Mahran A. Atef Fawzy M. Abdel Hafez S. M. N. (2022). Leukotriene receptor antagonist, montelukast ameliorates L-NAME-induced preeclampsia in rats through suppressing the IL-6/jak2/STAT3 signaling pathway. Pharm. (Basel) 15 (8), 914. 10.3390/ph15080914 PMC9332684 35893738

[B2] Ahmadian E. Rahbar Saadat Y. Hosseiniyan Khatibi S. M. Nariman-Saleh-Fam Z. Bastami M. Zununi Vahed F. (2020). Preeclampsia: microbiota possibly playing a role. Pharmacol. Res. 155, 104692. 10.1016/j.phrs.2020.104692 32070720

[B3] Alanazi A. S. Victor F. Rehman K. Khan Y. H. Yunusa I. Alzarea A. I. (2022). Pre-existing diabetes mellitus, hypertension and KidneyDisease as risk factors of preeclampsia: a disease of theories and its association with genetic polymorphism. Int. J. Environ. Res. Public Health 19 (24), 16690. 10.3390/ijerph192416690 36554576 PMC9778778

[B4] Arechvo A. Wright A. Syngelaki A. von Dadelszen P. Magee L. A. Akolekar R. (2023). Incidence of preeclampsia: effect of deprivation. Ultrasound Obstetrics Gynecol. 61 (1), 26–32. 10.1002/uog.26084 36178775

[B5] Atigan A. Tan S. Cetin H. Guler O. T. Ozdamar S. Karakaya Y. A. (2022). CD97 expression level and its effect on cell adhesion in Preeclampsia. BMC Pregnancy Childbirth 22 (1), 967. 10.1186/s12884-022-05280-z 36572878 PMC9791749

[B6] Awor S. Abola B. Byanyima R. Orach C. G. Kiondo P. Kaye D. K. (2023). Prediction of preeclampsia at St. Mary's hospital lacor, a low-resource setting in northern Uganda, a prospective cohort study. BMC Pregnancy Childbirth 23 (1), 101. 10.1186/s12884-023-05420-z 36755228 PMC9906950

[B7] Chen X. Li P. Liu M. Zheng H. He Y. Chen M. X. (2020). Gut dysbiosis induces the development of preeclampsia through bacterial translocation. Gut 69 (3), 513–522. 10.1136/gutjnl-2019-319101 31900289

[B8] Dimitriadis E. Rolnik D. L. Zhou W. Estrada-Gutierrez G. Koga K. Francisco R. P. V. (2023). preeclampsia. Nat. Rev. Dis. Prim. 9 (1), 8. 10.1038/s41572-023-00417-6 36797292

[B9] Gogoi P. Sinha P. Gupta B. Firmal P. Rajaram S. (2019). Neutrophil-to-lymphocyte ratio and platelet indices in preeclampsia. Int. J. Gynecol. Obstetrics 144 (1), 16–20. 10.1002/ijgo.12701 30362112

[B10] Govender V. Naidoo T. D. Foolchand S. (2023). The preeclampsia, growth restriction, and ductus venosus Doppler (GRADED) study: an observational study of early-onset fetal growth restriction and preeclampsia. Int. J. Gynecol. Obstetrics 161 (1), 106–113. 10.1002/ijgo.14495 36200937

[B11] Hallum S. Basit S. Kamper-Jørgensen M. Sehested T. S. G. Boyd H. A. (2023). Risk and trajectory of premature ischaemic cardiovascular disease in women with a history of preeclampsia: a nationwide register-based study. Eur. J. Prev. Cardiol. 30, 506–516. 10.1093/eurjpc/zwad003 36702629

[B12] Hodgman C. Khan G. H. Atiomo W. (2022). Coenzyme A restriction as a factor underlying preeclampsia with polycystic ovary syndrome as a risk factor. Int. J. Mol. Sci. 23 (5), 2785. 10.3390/ijms23052785 35269927 PMC8911031

[B13] Huang L. Liu Z. Wu P. Yue X. Lian Z. He P. (2022). Puerariae lobatae radix alleviates preeclampsia by remodeling gut microbiota and protecting the gut and placental barriers. Nutrients 14 (23), 5025. 10.3390/nu14235025 36501055 PMC9738998

[B14] Khan N. Andrade W. De Castro H. Wright A. Wright D. Nicolaides K. H. (2020). Impact of new definitions of preeclampsia on incidence and performance of first-trimester screening. Ultrasound Obstetrics Gynecol. 55 (1), 50–57. 10.1002/uog.21867 31503372

[B15] Litwinska M. Litwinska E. Bouariu A. Syngelaki A. Wright A. Nicolaides K. H. (2021). Contingent screening in stratification of pregnancy care based on risk of preeclampsia at 19-24 weeks' gestation. Ultrasound Obstetrics Gynecol. 58 (4), 553–560. 10.1002/uog.23742 34309913

[B16] Lu H. Q. Hu R. (2019). The role of immunity in the pathogenesis and development of preeclampsia. Scand. J. Immunol. 90 (5), e12756. 10.1111/sji.12756 30739345

[B17] Lv L. J. Li S. H. Wen J. Y. Wang G. Y. Li H. He T. W. (2022). Deep metagenomic characterization of gut microbial community and function in preeclampsia. Front. Cell Infect. Microbiol. 12, 933523. 10.3389/fcimb.2022.933523 36189343 PMC9515455

[B18] Malz S. S. (1946). Subacute hypoproteinemia in pregnancy. Harefuah 31 (9), 154–158. 20267120

[B19] Miao T. Yu Y. Sun J. Ma A. Yu J. Cui M. (2021). Decrease in abundance of bacteria of the genus Bifidobacterium in gut microbiota may be related to preeclampsia progression in women from East China. Food & Nutr. Res. 65. 10.29219/fnr.v65.5781 PMC8254465 34262418

[B20] Moodley M. Moodley J. Naicker T. (2020). The role of neutrophils and their extracellular traps in the synergy of preeclampsia and HIV infection. Curr. Hypertens. Rep. 22 (6), 41. 10.1007/s11906-020-01047-z 32462480

[B21] Negre-Salvayre A. Swiader A. Salvayre R. Guerby P. (2022). Oxidative stress, lipid peroxidation and premature placental senescence in preeclampsia. Archives Biochem. Biophysics 730, 109416. 10.1016/j.abb.2022.109416 36179910

[B22] Phoswa W. N. Khaliq O. P. (2021). The role of oxidative stress in hypertensive disorders of pregnancy (preeclampsia, gestational hypertension) and metabolic disorder of pregnancy (gestational diabetes mellitus). Oxidative Med. Cell. Longev. 2021, 1–10. 10.1155/2021/5581570 PMC8184326 34194606

[B23] Salma U. (2022). Relationship of serum lipid profiles in preeclampsia and normal pregnancy, Bangladesh. Afr. Health Sci. 22 (2), 475–479. 10.4314/ahs.v22i2.55 PMC9652639 36407348

[B24] Sun B. M. Meng L. Liu H. Bao D. (2020). Changes in intestinal flora in preeclampsia rats and effects of probiotics on their inflammation and blood pressure. Eur. Rev. Med. Pharmacol. Sci. 24 (19), 10155–10161. 10.26355/eurrev_202010_23235 33090423

[B25] Syngelaki A. Magee L. A. von Dadelszen P. Akolekar R. Wright A. Wright D. (2022). Competing-risks model for preeclampsia and adverse pregnancy outcomes. Ultrasound Obstetrics Gynecol. 60 (3), 367–372. 10.1002/uog.26036 35866878

[B26] Tesfa E. Munshea A. Nibret E. Mekonnen D. Sinishaw M. A. Gizaw S. T. (2022). Maternal serum uric acid, creatinine and blood urea levels in the prediction of preeclampsia among pregnant women attending anc and delivery services at bahir dar city public hospitals, northwest Ethiopia: a case-control study. Heliyon 8 (10), e11098. 10.1016/j.heliyon.2022.e11098 36303922 PMC9593197

[B27] Udenze I. C. (2016). Association of preeclampsia with metabolic syndrome and increased risk of cardiovascular disease in women: a systemic review. Niger. J. Clin. Pract. 19 (4), 431–435. 10.4103/1119-3077.180055 27251955

[B28] Vaught A. J. Boyer T. Ziogos E. Amat-Codina N. Minhas A. Darwin K. (2023). The role of proprotein convertase subtillisin/kexin type 9 in placental salvage and lipid metabolism in women with preeclampsia. Placenta 132, 1–6. 10.1016/j.placenta.2022.12.008 36603351 PMC13355416

[B29] Wind M. van den Akker-van Marle M. E. Ballieux B. Cobbaert C. M. Rabelink T. J. van Lith J. M. M. (2022). Clinical value and cost analysis of the sFlt-1/PlGF ratio in addition to the spot urine protein/creatinine ratio in women with suspected preeclampsia: PREPARE cohort study. BMC Pregnancy Childbirth 22 (1), 910. 10.1186/s12884-022-05254-1 36474150 PMC9727903

[B30] Yang X. Jiang R. Yin X. Wang G. (2022). Pre-BMI and lipid profiles in association with the metabolic syndrome in pregnancy with advanced maternal age. Contrast Media & Mol. Imaging 2022, 1–5. 10.1155/2022/4332006 PMC9288333 35854775

[B31] Yu Z. Liu Y. Zhang Y. Cui J. Dong Y. Zhang L. (2023). Ulinastatin ameliorates preeclampsia induced by N(gamma)-nitro-l-arginine methyl ester in a rat model via inhibition of the systemic and placental inflammatory response. J. Hypertens. 41 (1), 150–158. 10.1097/hjh.0000000000003316 36453658

[B32] Zheng H. Mai F. Zhang S. Lan Z. Wang Z. Lan S. (2023). *In silico* method to maximise the biological potential of understudied metabolomic biomarkers: a study in preeclampsia. Gut 73, 383–385. 10.1136/gutjnl-2022-329312 36725314

